# The challenge of cytotoxic T cell responses in carcinoma with a focus on lung carcinoma

**DOI:** 10.3389/fonc.2025.1669230

**Published:** 2025-12-17

**Authors:** Aditya Dash, Afsheen Banisadr, Donivian Al Dikka, Scott C. Johns, Mark M. Fuster

**Affiliations:** 1 VA San Diego Healthcare System, San Diego, CA, United States; 2 Division of Pulmonary, Critical Care, and Sleep Medicine, University of California, San Diego, La Jolla, CA, United States; 3 Veterans Medical Research Foundation, San Diego, CA, United States

**Keywords:** cancer, cytotoxic T-lymphocyte, immunity, T cells, tumor microenvironment, vaccine, lung cancer

## Abstract

Immunity during cancer progression involves critical responses that may be harnessed to augment anti-tumor cytotoxicity. A potent arm of immunity in cancer involves cytotoxic T cells (a key CD8+ T-lymphocyte subset): Anti-tumor responses by such cells optimally involve sensitization and acquired responses to tumor antigens by antigen presenting cells. Many tumor microenvironment (TME) biophysical and functional limitations in carcinomas limit robust achievement of this ideal cellular-immunologic pathway. This is especially challenging in lung carcinoma, on which we focus mechanistically and with an eye to therapeutic translation. Localization of tumor-sensitized and activated CD8+ T cells to tumor “nests” with efficient tumor cytolysis involves many challenging steps. Amplifying and sustaining such responses is also a unique challenge. The variety of homeostatic and immunosuppressive obstacles often becomes overwhelming. Measuring the state of this response during lung cancer progression is also challenging, making it difficult to mount (and/or predict) T-cytotoxic responses in the heterogeneous and dynamic carcinoma antigen landscape. We investigate these challenges herein, while examining strategies to boost T-cytotoxic immunity in lung cancer through novel and emerging immunotherapeutic approaches. Beyond current immune checkpoint blockade approaches that are relatively non-specific with respect to antigen recognition by the T-cell receptor, we highlight ongoing and translational vaccines, cell-therapies, antigen-presenting cell boosting approaches, T-cell therapies, and biophysical considerations with an eye to overcome key barriers of this powerful arm of anti-tumor immunity.

## 1 Introduction: acquired cellular immunity in carcinoma and the cytotoxic T cell

Cytotoxic CD8+ T cells in cancer are a key arm of acquired anti-tumor immunity that is highly effective when empowered under ideal conditions in the tumor microenvironment (TME). A variety of barriers to the ideal host-response to tumor formation and growth introduce major challenges along with several opportunities from a clinical and translational perspective. In addition to a balance of effector and suppressive immune mechanisms at play while tumor “accessible” host dendritic and effector T cells respond to cancer-associated epitopes, the TME challenges such responses from additional biophysical, vascular, metabolic, and other standpoints (1–3). Understanding why a host is unsuccessful in mounting an adaptive cellular immunologic response against a tumor is vital for developing effective immunotherapies, but the nature of such limitations varies among tumor subtype and host TME (2, 4, 5). Elucidating these limitations and the major mechanisms involved is critical for empowering cellular adaptive immunity in distinct cancer- and patient-specific contexts. From efforts to augment endogenous cancer antigen presentation and improved “Signal-1” antigen recognition to new vaccine and engineered cell therapies to better manipulate immune checkpoint responses as well as adapter/engager mechanisms along with other strategies, we consider advances in boosting endogenous and exogenous forces that empower CD8+ T cell immunity in the carcinoma setting with an eye to unique challenges in lung cancer.

From a general perspective of acquired immunity in cancer, the system of acquired immunity evolved to distinguish foreign antigens from self and mount a response to eradicate pathogens. In cancer, the ability to mount a cellular response to a “foreign threat” is more complicated. The lack of consistent expression of non-self (i.e., neo-antigen) epitopes in a uniform manner across all or most neoplastic cells in carcinoma is a major impediment. However, even when cancer-expressed neo-epitopes are broadly expressed in subsets of carcinoma cells within the tumor, the ability to mount robust acquired cellular immune responses against a heterogeneous cellular landscape remains limited (6, 7). Part of this challenge stems from the fact that carcinoma evolves as a transformation of host epithelial tissue, resulting in predominant expression of normal host-cell epitopes (2). This leads to multiple (poly-clonal) epitopes are not abundantly or broadly expressed, a few (dispersed) mutation-driven neo-epitopes, and generally a highly variable antigen landscape across the carcinoma mass (8). Furthermore, immune evasion in cancer may be driven by homeostatic responses that activate regulatory “brakes” on host effector immune cells (9). As a result, the immune system’s ability to respond to a carcinoma may be restricted to a limited epitope repertoire expressed on a limited number of carcinoma cells at any given moment in the macroscopic tumor (6, 10).

### 1.1 In the TME, the presence of CD8+ T-cells is crucial in mounting an acquired immune response against tumors

When serving as sensitized effector cells engaging in anti-tumor cytolytic activity against tumor cells, CD8+ T-cells are referred to as cytotoxic T lymphocytes or CTLs (11). They are a functional subset of the more general tumor-infiltrating lymphocyte (TIL) population of cells. Outside of cancer, such cytotoxic cells may activate and expand in response to any of a variety of cellular antigen targets, which may include host cells with intracellular infection by unique bacteria or viruses, for example (12). The classical mechanism by which such responsive CD8+ T-cells function includes initial priming by dendritic cells as “professional” antigen-presenting cells (APCs). Alternative APCs include macrophages or B cells. In such non-tumor scenarios, there is the challenge of expanding multiple primed CTLs with distinct specificities to “stay ahead” of the antigen-expressing pathogens as they infect host cells. In cancer, tumor-cell nests may pose a similar challenge that paces ahead of immunity as tumor heterogeneity rapidly evolves (6, 8, 10). Microscopically, such tumor-cell compartments may be associated with physical barriers at the tissue or matrix level. This is among several other factors such as repressive immunologic counter forces (e.g., cytokines or growth of suppressive cell subsets) and/or tumor cell-surface shielding and repression by tumor glycocalyx as obstacles (2, 9, 10). We herein use the term “CD8+ T cell” to refer more generally to either naïve or sensitized anti-tumor effector T cells (CTLs) – where inhibitory pressures in the TME may limit naïve CD8+ T cell activation or CTL functions. We also generally use the term “effector” to refer to immunologic cells with anti-tumor functional capacity.

### 1.2 Empowering the CTL in a dynamic and repressive TME

Ideally, translational advances may facilitate endogenous expansion robust effector CTLs at the “site of action” to eradicate carcinoma cells within a highly dynamic carcinoma antigen landscape. Two unique challenges in the setting of carcinomas that make this very difficult to achieve include the fact that: (1) Tumors often over-express self-antigens that do not drive antigen-presenting cells (APCs) to cross-present antigen to naïve T cells; and (2) Even with constantly changing neo-epitopes in distinct spatial regions of a growing tumor, homeostatic and tumor-specific immune-repressive or tolerance forces begin to dominate over time (6, 13, 14). The antigen landscape of carcinomas is thus elusive, even if the cellular immunologic “substrates” (i.e., APCs and T cells) are in the right place at the right time. In general, a better clinical outcomes are associated with the histologic presence of T cells at the site of the cancer cell nests (i.e., not shielded by tumor stroma) (2). If this occurs relatively early following transformation or tumor growth, it follows that a more robust anti-tumor response may inhibit tumor progression. Immune exhaustion, repression, and tumor immune-escape mechanisms dampen effector CD8+ T cell functions if early and robust anti-tumor responses are not successful (15). Importantly, considering anti-tumor T cells as a class, the CD4 subset often facilitates CD8+ responses, but similar challenges exist for such cells when functioning as effectors in the dynamic cancer TME. Such CD4+ subsets also evolve into T-regulatory or suppressor cells that impair effector responses in carcinomas (14). More generally, such cells may also be essential in key “helper” functions potentiating anti-tumor responses by B cells and natural killer (NK) cells in the TME (16).

## 2 Barriers to anti-tumor cellular immunity in lung cancer

### 2.1 Introduction to cellular immune kinetics and barriers in the lung cancer TME

In carcinomas, TILs become sensitized to elicit specific anti-tumor cytolytic responses as CTLs (11) in the primary tumor or in metastatic and/or lymph node draining sites (17). In lung carcinoma as a particularly aggressive tumor in terms of early metastasis, even if TILs are appropriately tumor-sensitized and activated, they must migrate through unique physical barriers imposed by a lung-unique TME. Moreover, distinct families of chemokines in the lung cancer TME variably drive migration patterns of TILs, with unique responses to chemokines (along with stimulatory and repressive cytokines) released from tumor cells, myeloid (e.g., macrophage), or lymphatic vasculature, depending on the carcinoma sub-type (18–20). Despite the presence of immune activating signals and tumor-directed chemotactic stimuli, stromal barriers may significantly hinder ultimate effector responses (10).

In some cases, a paucity of intra-tumoral pro-migratory chemokines may further limit effector T cell entry and activation, while a more dense extracellular matrix may impair TILs (including naïve and sensitized CD8+ T cells) from reaching dense regions of cancer cells. Occasionally, fibrotic desmoplasia responses - frequently present in acinar-pattern invasive adenocarcinomas - may markedly limit T cell infiltration, and this may progress while immunologic escape takes place within the tumor stroma (1, 3). Theoretical forces that may limit such responses by the remodeling ECM include the expression of hyaluronidase or collagenase or even anti-fibrotic agents that can digest collagen or hyaluronan in lung tumor matrices (1, 21, 22). This may facilitate T cells in their ability to reach tumor cellular regions (23). The potential for carcinoma cells to spread under the same matrix remodeling conditions, however, must also be considered. Indeed, in some cases tumor cell secretion of hyaluronidase or even heparinase can facilitate the tumor’s tissue-invasive capacity while remodeling surrounding matrix elements (i.e., hyaluronan or heparan sulfate), and facilitating invasion/metastasis along with vascular responses (24, 25). Nevertheless, ECM barrier challenges, including novel approaches to promote immune penetration of the tumor’s fibrous stroma, remain a concept that can be explored in the development of host-modulating strategies to promote anti-tumor immunity (26).

### 2.2 Stromal cell remodeling and the progression of effector CD8+ T cell barriers in carcinomas

**Figure 1:**
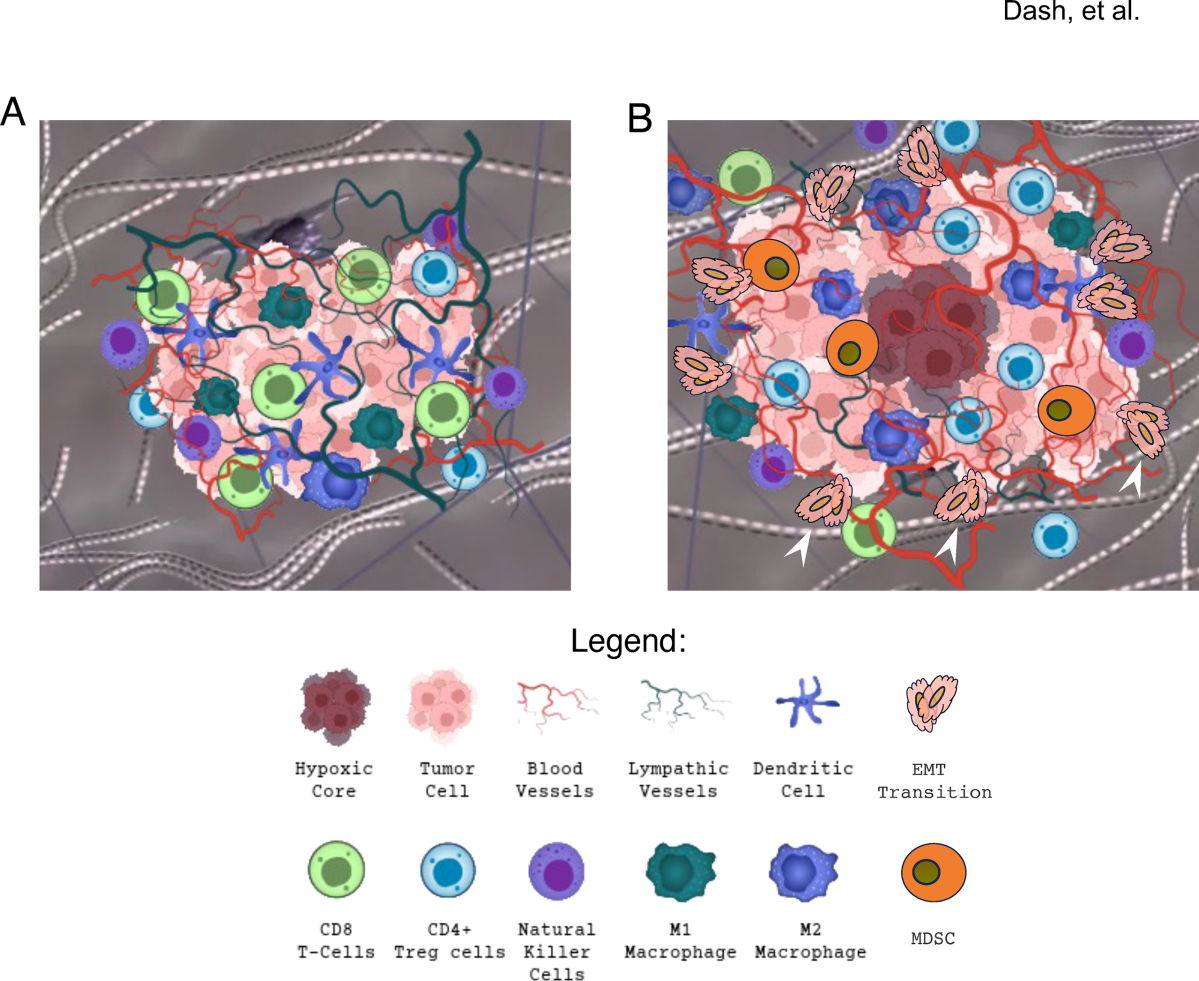
Evolution in immunity effector to exhaustion states in the lung carcinoma microenvironment. A major theme in the evolution of myeloid-derived cells in the lung cancer TME in general is the transition from early effector responses to a combination of homeostatic and active repressive forces as the TME evolves during continued tumor growth, hypoxic and anaerobic metabolism, and the influence of a variety of host cellular and tumor stromal transitions. The drivers for this are discussed in detail in respective sections of the main text, while we here illustrate initial themes in cellular transitions that ultimately challenge continued effector responses and promote tumor immunologic escape. **(A)** During early adaptive immunity by both naïve and sensitized CD8+ T cells in a growing primary lung carcinoma, angiogenic growth is paralleled by clonal CD8+ T cell expansion and anti-tumor cytolytic responses after appropriate antigen sensitization (i.e., CTL cells), along with effector dendritic cell augmentation as well as NK cell and macrophage infiltration. While the latter include subsets of M1 inflammatory macrophages that also facilitate anti-tumor cytokine and effector responses, further tumor remodeling, including rapid anaerobic growth and hypoxic region development as well as tumor-cell epithelial-to-mesenchymal (EMT) transitions (white arrowheads) drive tumor invasion and metastasis. These events are associated with suppressive immunologic evolution as illustrated in: **(B)** This state is characterized by transitions in cytokines and the evolution and influx of suppressive immunologic cell subsets that drive CD8+ T cell exhaustion (increased expression of co-inhibitory PD-1, TIM3, LAG3), including CD4+ T cell subsets dominated by T-regulatory (Treg) cells and a paucity of CTLs or M1 macrophages. With growth of pathologic angiogenic vessels, tumor interstitial pressure increases with absence of deep intra-tumoral lymphatic vessels and hypoxic progression. The state is also impacted by increases in myeloid-derived suppressor cells (MDSCs) and expansion of M2 subtype macrophages with production of immunosuppressive cytokines such as TGF-β and IL-10. Production of VEGF and IL-6 by M2 macrophages further promotes tumor growth and EMT.

Beyond a variety of physical barriers and limitations in the ability of a naïve or sensitized CD8+ T cell to “navigate” the TME stroma and ECM in the setting of cancer, stromal remodeling brings further limitations to cellular immunologic success. One challenge is the ability of carcinomas to inhibit CTLs from effectively detecting tumor cells upon near-contact, inhibiting CTL expansion, and resulting in attenuated anti-tumor responses. This ability may be fueled by a variety of tumor- and host-driven responses that disguise tumor cells from host-effector immune responses at the molecular level (2). Epigenetic alterations, for example, can change the efficacy of transcription factors, altering the expression of genes that regulate immune evasion and tumor heterogeneity. This may limit carcinoma antigen recognition in distinct solid tumor domains. The CD4 T cell subset may play critical roles in modulating such responses in the setting of repeated immunogenic challenges, and while CD4+ helper functions may promote anti-tumor responses by evolving CTL cells in the tumor, the development and expansion of a T-regulatory (Treg) subset of such cells can drive tolerance and suppression of anti-tumor immunity (10). Moreover, dysregulated transcription factor expression can render the functions of these cells ineffective, which in turn may inhibit the actions of natural killer (NK) cells as well as CTL functional responses (27). The impact of these responses limits pathways that drive the formation of both memory and effector T cells, and facilitates cancer survival (28). **Figure 1** illustrates how multiple T cell subsets, in addition to CTLs, dendritic APCs, and subsets of macrophages and NK cells variably promote anti-tumor cellular immunity, while suppressive forces within the same tumor by distinct T cell and macrophage subsets, myeloid-derived suppressor cells (MDSCs) (29). This promotes evolution of a tolerogenic cytokine milieu and a parallel exhaustion immuno-phenotype.

## 3 Major spheres of cytotoxic T cell suppression in lung cancer

### 3.1 Challenges in augmenting the specificity and magnitude of cellular immunity in lung carcinoma

Among malignant tumors, carcinomas, deriving from the transformation of epithelial tissues, are particularly challenging for the immune system to recognize and destroy. Carcinomas are a common malignancy originating from epithelial cells of the skin, lungs, breast, colon, pancreas, prostate, and/or other organs. Among carcinomas, the diversity of tissues from which they originate may also impact the ability to generate CD8+ T cell responses. With this in mind, a variety of insights have grown from harnessing T cell immunotherapeutic approaches to promote tumor cytotoxic responses by modifying immune checkpoint pathways, or translation in employing engineered T cells among several such neoplasms (30). For example, in lung cancer among other epithelial-cell tumors, the tumor mutation burden (TMB) is often considered a reasonable index of CD8 T cell responsiveness to a unique antigen load expressed by tumor cells. Malignancies arising from intense environmental mutagenic drivers (characterized often by higher TMB indices) such as melanoma/squamous skin carcinoma (mutagenesis from sun exposure), or squamous-cell lung cancer (driven by tobacco mutagens) typically drive greater CD8+ T cell responses (2, 8). Despite these forces, repressive effects by Treg cells and non-inflammatory macrophages, among other cells ultimately represent a cell-immunologic “sphere” that dampens these effector responses.

Even in settings of a relatively high TMB, while nonspecific activation of T cell anti-apoptosis pathways may help to activate and expand CTLs (e.g., immunotherapy using blocking antibodies against the T cell immune checkpoint receptor PD1), if robust MHC-I/antigen recognition by the CTL’s T cell receptor (TCR) is lacking, then responses to such immunotherapy may be limited. In parallel to the specificity of this key Signal 1 immunologic event, additional harnessing co-stimulatory pathways or further blocking co-inhibitory pathways (e.g., with bispecific anti-checkpoint inhibitors) becomes appealing (31, 32). Interestingly, unique complex carbohydrates (glycans) also regulate antigen presentation in a variety of carcinomas, including lung cancer. In pre-clinical studies, targeting the sulfation or charge of such glycans on APCs may serve as a strategy to boost antigen presentation to naïve T cells and promote CD8+ TIL infiltration *in vivo* (33, 34). Extensions of that work point to similar behavior by lymphatic endothelial cells. Curiously, such mesenchymal-derived cells may serve as alternative antigen presenting cells in the TME (35). Much more work is needed to understand how to augment presentation of tumor antigens to CD8+ T cells by such novel APCs in the TME, including coordinated downstream CD4+ and CD8+ anti-tumor responses in the setting of lung cancer progression.

### 3.2 Metabolic drivers of impaired anti-tumor CD8+ T cell function in lung carcinoma

We may conceptualize another sphere of CD8+ T cell repression to function from the standpoint of a number of metabolic inputs that essentially “toxify” the CD8+ T cell’s ability to activate and sustain anti-tumor cytotoxic functions in the carcinoma microenvironment. These include the state of hypoxia, glucose and lactate metabolism, and depletion of key amino acids that play rate-limiting roles in T cell metabolism and survival (36). Tumor hypoxia is a central player in several metabolic derangements that impair or exhaust effector CD8+ T cell functions in the TME. Hypoxia occurs frequently in tumors, often resulting in central necrotic zones where demand from tumor metabolic rates and growth exceed the tumor blood supply carrying oxygen to affected regions. Hypoxia forces a shift away from mitochondrial ATP production, while tumor cell anaerobic metabolism is facilitated by rapid tumor cell glucose uptake as a result of HIF-1α induced up-regulation of glucose transporters. This greatly increases local lactate production. Even when oxygen is present, carcinoma cells are endowed with a propensity to preferentially produce lactate through glycolytic glucose metabolism (the Warburg effect). This rapidly generates lactate, which serves as a way to rapidly regenerate NAD+ from NADH and pyruvate via LDH (37): In turn, this is essential for continuation of the glycolytic pathway, and further perpetuates it. Interestingly, recent targeting a major LDH enzyme subtype (LDHA) through inhibitor approaches can deplete these substrates, and drive carcinoma-cell apoptosis as a proof-of-concept (38). With specific regard to amino acid metabolism, tumor overproduction of the enzyme indoleamine 2,3-dioxygenase (IDO) depletes critical supplies of tryptophan, leading to amino acid starvation and anergy of sensitive T cells (36). At the same time, while the more abundant plasma amino acid glutamine is rapidly utilized to maintain key metabolite pools in cancer cells, its limitation in T cells may impair activation associated growth and proliferation (39).

Elevated lactate production in the carcinoma TME is particularly detrimental to CD8+ T cells. Along with a glucose “steal” by neighboring tumor cells that out-competes cytotoxic T cells (and NK cells as well as DCs) for glucose, rapid lactate production inhibits both CTL survival as well as NK cell cytokine production and cytotoxicity (36). Interestingly, Tregs may thrive in parallel given their lower glucose dependence (40), while up-regulation of GLUT transporters in some repressive cells (especially MDSCs) can further drive the ability of such cells to out-compete local TME effector CD8+ T cells for glucose (41). Moreover, lactate drives suppressive M2 macrophage polarization, IL-10 production, and MDSC infiltration while inhibiting DC differentiation (42, 43). With respect to macrophage functions, lactate accumulation in the TME may directly induce “lactylation” of histone proteins, altering transcription factor expression in carcinoma cells and promoting the M2 phenotype (44, 45). Lactate also drives unique G-protein coupled receptor expression, including up-regulation of Gpr81 as a lactate receptor itself via STAT3 signaling in lung cancer cells (46). In addition to effects on macrophages and other immunologic cells, this suppresses CTL functions by directly driving lung cancer cell PD-L1 production (44, 47). These are among several important metabolic mediators that facilitate creation of an immune-suppressed TME in addition to directly inhibited CTL functions.

### 3.3 The vascular compartment and hypoxia

Carcinomas in general evolve macroscopically to variable extents in the degree to which tortuous vascular infiltration of the tumor (angiogenesis) and tumor cell growth out-pace the ability to relieve tumor interstitial pressure and lymphatic drainage (remodeled during lymphangiogenesis) (63, 64). These dynamics partly contribute to a tumor “core” with necrosis and hypoxia as blood flow becomes limiting, and deep lymphatics are compressed and/or absent in lung carcinoma, while the level of lymphangiogenesis (driven by tumor production of VEGF-C) correlates with lymph node metastasis (65). While physical limitations to CD8+ T cell infiltration exist in this setting, leaving large regions of the tumor with greater immunologic “access” challenges, signaling by HIF leads to markedly elevated VEGF production along with the release of FasL, both of which promote CD8+ T cell apoptosis in the necrotic core (64, 66). This microenvironment is also associated with greater mitochondrial reactive oxygen species (ROS) release as well as greater expression of the PD-L1/PD-1 axis, promoting CD8+ T cell apoptosis and repression (52, 66). In lymphatic vasculature of the TME, MHC-I expression and tumor antigen display capacity paired with reduced co-stimulatory machinery and PD-L1 expression induces tolerance by CD8+ T cells making contact with tumor lymphatic vasculature (53, 54). Finally, in the hypoxic microenvironment associated with rapid tumor growth and anaerobic metabolism with lactate production (particularly in the tumor necrotic core) directly inhibits NK and CD8+ T cell functions, as discussed in **3.2** (36). This combination of vascular remodeling and tumor hypoxia associated features during lung cancer progression thus represses CD8+ T cell functions through a variety of unique mechanisms.

### 3.4 Suppressive cancer cell - CD8+ T cell contacts: glycan shielding and glyco-immune checkpoints

In lung cancer among several other carcinomas, unique features of the glycan layer coating the tumor-cell surface (or “tumor glycocalyx”) mediate suppression of neighboring NK cells and CD8+ T cells. This takes place through a variety of “shield” functions by the cancer cell glycocalyx, and these are particularly relevant in lung cancer, brain tumors, ovarian cancer, breast cancer, and a variety of other neoplasms. Heavy glycosylation by sulfated (heparan sulfate) and non-sulfated (hyaluronan) glycosaminoglycans as well as sialic acids creates a negatively charged glycocalyx layer above the cancer plasma membrane (67). Sialic acid in particular is heavily expressed on the distal “tips” of glycans that decorate tumor-cell glycoproteins and glycolipids (68). Direct sialic acid contact with Siglec-7 and Siglec-9 on NK cells as well as CD8+ T cells drives apoptotic signaling via siglec-mediated phosphorylation of Immunoreceptor Tyrosine-based Inhibitory Motifs (ITIM), which recruit phosphatases such as SHP-1 that dephosphorylate critical signaling molecules involved in activating NK or CD8+ T cells (57, 69).

Tumor cell sialic acid overexpression on key ligands for the NK cell activating receptor NKG2D (such as MICA/B or the ULBP family of ligands) is able to repress not only NK cell activity but also CD8+ T cells for which NKG2D also serves as a key co-stimulatory molecule. Strategies to inhibit or disrupt the carcinoma cell glycocalyx, and in particular sialic acid expression (70–73), may serve critical roles in blocking siglec activation and promoting NKG2D activation by infiltrating CD8+ T cells as well as NK cells that may critically promote anti-tumor immunity in lung cancer. These considerations are particularly key in the case of small cell lung cancer (SCLC), the most aggressive form of lung cancer (~15% of cases), where the sialic acid **r**epressive “don’t touch me” glycocalyx can be decorated with heavy sialic-acid rich gangliosides and even poly-sialic acid (polymers of α2,8 linked sialic acids) decorating key SCLC surface molecules such as CD24 and CD56 (74, 75). Overcoming this repression may be a key to augmenting anti-SCLC CD8+ T cell and NK cell immunity.

**Figure 2:**
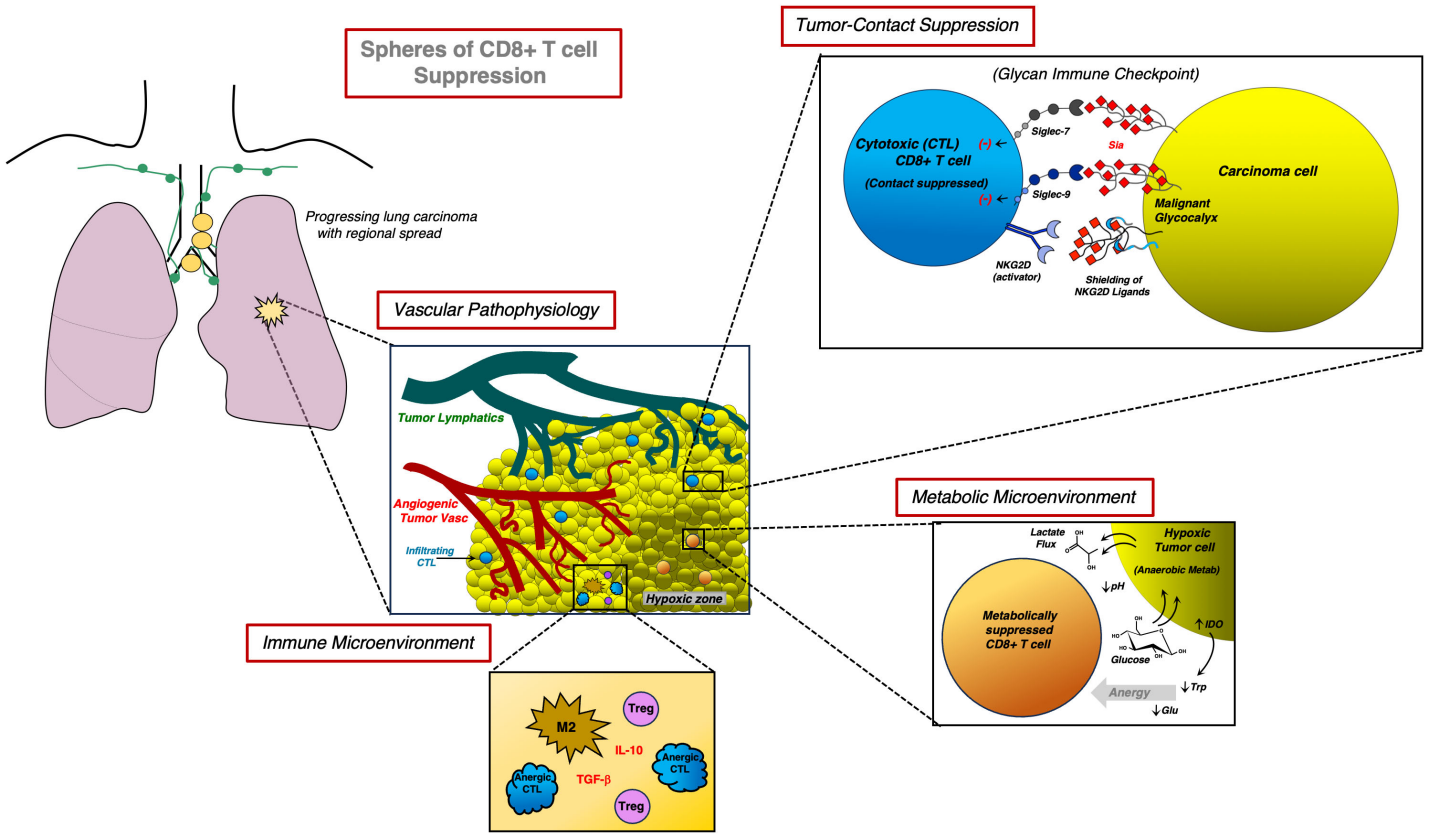
Key mechanistic spheres of CD8+ T cell suppression in the lung cancer immune microenvironment. In an advanced lung carcinoma illustrated as a cartoon (upper left) by a tumor in the left lung (yellow) with enlarged intrathoracic lymph nodes along the mediastinal lymphatic chain, a variety of forces drive repression of CD8+ T cells, which further promotes escape from adaptive anti-tumor immunity and tumor progression. During vascular remodeling of macroscopic tumor with early infiltration by cytotoxic CD8+ T cells in blue (sparse within detailed tumor; center inset), tortuous and aberrant angiogenic vasculature (red) lacking pericytes impairs systemic CD8+ T cell infiltration. Lymphatic vasculature (green) relegated to the tumor periphery is unable to relieve high intra-tumoral interstitial pressure. The summation promotes tumor hypoxia, anaerobic metabolism (lower right inset; metabolically suppressed CD8+ T cells in orange) along with a variety of events that increase cancer cell glucose uptake with rapid TME lactate production (Warburg effect), starvation of key tumor amino acids such as tryptophan (Trp; due to IDO generation) or glutamine (Glu), and further promotion of T-cell (including CTL) anergy. Despite influx of a variety of host effector cells, with further immunologic exhaustion (bottom inset), immunosuppressive M2 subtype macrophages and Treg cells expand and predominate with the release of cytokines that drive tolerance (e.g., TGF-β, IL-10) and further CTL anergy in the TME. A challenging additional impediment due to a suppressive glycocalyx “shield” (upper right inset) can contribute to further CD8+ T cell repression in a variety of lung cancers, particularly in SCLC, which often is characterized by heavy glycocalyx sialic acid (Sia) on tumor-surface gangliosides or even polysialic acid decorated surface proteins such as CD56. This terminal glycan shielding makes the ability to gap the CTL: Tumor-cell synapse and achieve tumor plasma membrane contact by CTL or NK cells especially difficult: Sia binding to Siglecs (especially 7 and 9) inhibits CTL activation. Moreover, Sia masking of key ligands for the activating NKG2D receptor on CTL and NK cells further represses cellular immunity, thwarting even the achievement of “final steps” in tumor cytolytic events by competent sensitized CD8+ T cells.

**Figure 2** illustrates several key themes highlighted in the above CD8+ T cell Spheres of Suppression reviewed in **Table 1**. From the unique lung cancer vascular environment with associated hypoxia and metabolic stress to a unique tolerance-inducing immunophenotype with glycocalyx shielding and CD8+ and NK cell repression by the lung cancer-cell glycocalyx, the various drivers of suppression promote tumor progression and invasion and metastasis. Current immunomodulating approaches and novel strategies under development discussed below (Section 5) are underway to overcome such obstacles, and empower anti-tumor cellular immunity.

**Table 1:** Spheres of CD8+ T cell suppression and key functional impacts in the TME.

Sphere of CD8+ T cell suppression	Functional impacts
Metabolic microenvironment Refs (36, 39–41, 44, 47, 48):	- Competing glucose substrate utilization by rapidly expanding carcinoma cells. - Lactate synthesis and tumor reliance on anaerobic glycolytic pathway: Associated suppression by M2 macrophages; Upregulation in tumor PD-L1 production and CD8+ T cell apoptosis - Depletion of critical amino acids such as tryptophan (IDO overproduction) and glutamine.
Immunologic microenvironment Refs (10, 14, 17, 43, 49, 50):	- Treg cell expansion and promotion of tolerance conditions and cytokine environment (including IL-10 and TGF-β release) - Anti-inflammatory M2 cytokines and growth factors, promoting additional tumor cell growth - Epigenetic modifications: Acetylation and methylation of specific oncogenes and tumor-suppressor genes promoting tumor progression
Vascular pathophysiology and hypoxia Refs (37, 51–55):	- Tumor angiogenic vessels impede CD8+ T cell delivery to the tumor, and induce suppression (via PD-L1), FasL release (CTL apoptosis), along with inhibitory effects from TME VEGF production - Hypoxic conditions with creation of zones of necrosis (worsened by augmented tumor interstitial pressure) drive anaerobic metabolism and CD8+ T cell suppression under high lactate production - Lymphatic vascular endothelium promotes antigen tolerance by trafficking CD8+ T cells - Hypoxic induction of Reactive oxygen species further promotes tumor vascular remodeling; requires sufficient balancing with antioxidant (e.g., GSH) buffering to support optimal CD8+ T cell functions. Superoxide is particularly CTL inhibitory
Carcinoma-cell contact suppression Refs (56–62):	- Carcinoma glycocalyx and tumor-surface sialic acid expression: Direct immune-cell repression via Siglec receptors on CD8+ T cells (and NK cells) - Carcinoma cell co-inhibitor ligands (e.g., PDL1- binding to PD1; Galectin-9 binding to TIM3) - Tumor sialic acid masking of ligands for NKG2D activating receptor on CD8+ T cells (and NK cells)

## 4 Unique lung histologic and physiologic features impacting anti-tumor lung cancer immunity

### 4.1 Histologic and microanatomic features and associated patterns of cytotoxic T cell immunity

Lung cancer remains the leading cause of cancer death in the U.S. and worldwide (76). Lung cancer cases remain steady in terms of overall incidence, while adenocarcinoma, the most common subtype of non-small cell lung cancer (NSCLC) has been on the rise among women and nonsmokers (77, 78). A variety of non- or minimally invasive lung adenocarcinomas, including pure adenocarcinoma *in situ* (AIS), and minimally invasive adenocarcinoma (MIA), are forms that result in slow, lepidic growth, while atypical adenomatous hyperplasia (AAH) is a pre-malignant state (likely in its earliest histologic form) (79). Invasive forms of adenocarcinomas have a poorer prognosis than non-invasive subtypes (i.e. AIS, MIA, AAH) despite best surgical or combined therapeutic approaches. The ability to characterize the CD8+ T cell response in resected specimens and biopsies has been valuable as a biomarker for indicating the pathogenicity of the adenocarcinomas (32, 80). Phenotypic evaluation of CT8+ T cells may also be a useful method for characterizing distinct cancer grades by cellular immune status. More work is needed to develop assays that characterize patient-specific CTL capacity, including real-time functional testing from fresh biopsy specimens. This may drive the application of unique CD8+ T cell augmentation platforms as immunotherapy advances are made in lung cancer.

### 4.2 Lung physiologic features of neoplasia progression and cell-immunologic impacts

Carcinomas and their treatment also affect lung function, including airway patency as well as gas exchange, which can drive processes such as post-obstructive infection, lobar collapse, lung inflammatory and fibrotic responses, and hypoxemia. In particular, beyond morbidity and mortality as a result of distant metastasis complications, the local spread of lung carcinoma can also impair respiratory function both directly (alveolar and lymphangitic spread as well as airway compression and bleeding), or because of therapy-induced side effects. The latter include inflammatory responses to drugs and/or the physiologic effects of lobar lung resection or radiation therapy. Airway compression and bleeding become even more threatening with centrally aggressive lung carcinomas such as squamous cell lung cancer, characterized by a higher TMB and immunotherapeutic responsiveness (i.e., to inhibitory checkpoint blockade), or small cell lung cancer, which has more limited immunotherapeutic responsiveness (81). Tumor histology may thus drive distinct immunologic patterns irrespective of anatomic progression; however, one should consider the possibility that local complications may modify the immunologic landscape as a result of new inflammatory events, infection, or even central tumor necrosis and hypoxia (82). Empowering CTL cells through checkpoint blockade can also lead to immune-related adverse events (IRAE), which present in a subset of cases as autoimmune responses in a variety of organs (with pneumonitis as a particularly serious complication) as a result of off-target CD8+ T cell activation. This highlights a key example of the challenge with specificity in immune augmentation (83).

### 4.3 Lung neoplasia and uniquely associated driver mutations affect anti-tumor cellular immunity

It is instructive to consider CD8+ T cell responsiveness to current clinical immunotherapy along with the challenge of unique lung cancer mutations that both guides targeted and first-line therapy while significantly modifying CTL responsiveness and ultimate prognosis. This is an important concept in advanced-stage non-small cell lung cancer, where the presence of certain driver mutations critically determines the primary therapeutic step. The frequency of such mutations (e.g., targetable EGFR versus KRAS mutations) among lung cancer patients varies by global region and tobacco exposure. In general, the presence EGFR or KRAS mutations (as the most encountered ones) correlate with lower PD-L1 or PD-1 expression, respectively (84). However, KRAS mutations are more likely to occur in the setting of tobacco exposure, and are associated with a greater tumor mutation burden along with a greater incidence of an immunologically “hot” TME. Their presence is also associated with greater responsiveness to immune checkpoint blockade (85). On the other hand, EGFR mutant lung cancer is more immunologically “cold,” and while treatment with higher-generation targeted therapy (e.g., Osimertinib) is associated with greater overall survival and improved prognosis, immune checkpoint blockade responsiveness by such tumors is low (85).

## 5 Boosting cellular immune therapeutic avenues in lung cancer - empowering the CD8+ T cell

Currently approved immune checkpoint inhibition (ICI) approaches in lung cancer, including approaches that pair ICI with cytotoxic therapy, have been transformative in a subset of patients with advanced and metastatic disease (86). Beyond this basic approach which centers mostly on antibody blockade of the PD-1/PD-L1 T-cell repressive axis, avenues for promoting CTL effector responses in the lung cancer microenvironment are limited. Ongoing investigation examines a variety of promising strategies that probe various pathways to augment effector CD8+ T cell presence as well as key antigen-specific and non-specific co-stimulatory mechanisms at the immune synapse in lung cancer. Along with a renewed effort in establishing novel lung cancer vaccine approaches and adoptive cell therapies (ACT), including engineered chimeric antigen receptor T (CAR-T) cell strategies, a variety of novel bispecific T-cell engager molecules, chemokine strategies, and new ICI targets are driving new immunologic discoveries (86–89). This is with a mind to augmenting the actions of endogenous anti-tumor CD8+ T cells despite challenges of tumor antigenic clonal variation within a highly dynamic antigen landscape and a limited lifespan for newly generated CTLs (before exhaustion) (8, 90). These avenues also involve augmenting CTL function and effector T cell expansion in the TME in parallel with other therapeutic strategies. Current use of ICI is a very active and effective state-of-the-art therapy in ~25% of cases of advanced-stage NSCLC. It is increasingly paired with surgical neoadjuvant/adjuvant approaches to improve outcomes in earlier-stage lung cancer in recent trials, advancing the standard of care (86, 91).

### 5.1 Lung cancer vaccine innovation and the CD8+ T cell

Cancer vaccines have significant potential due to their theoretical efficacy, relative safety, convenience, and broad potential for applicability to a variety of carcinomas. In lung cancer, while they have not made their way to routine clinical use (92), a variety of new strategies are being examined in recent and ongoing studies, including broad platforms from cell-based (antigen-modified autologous dendritic cells or whole tumor cells), to peptide or viral vector-based platforms to nucleic-acid (including mRNA) platforms (87, 93). Vaccines can be divided generally into either: (1) tumor specific antigens (TSAs) designed from neoantigen encoding mutations or oncoviral antigens; or (2) tumor associated antigens (TAAs) designed from self-proteins over-expressed by cancer cells (87, 94). An important consideration in both novel vaccine and cell therapy innovations, however, is that evolution in the design of more patient-centric or “personalized” vaccines or adoptive/engineered T-cell constructs comes with limitations in cost and scalability. It is critical to factor this into patient/platform design.

Several lung cancer vaccines have often employed peptides that are efficient in sensitizing and activating antigen presenting cells (APCs), often paired with a potent adjuvant agent while the most advanced (mostly phase I/II) in development at this time have been delivered as short as well as long peptide constructs (87). Immunogenic glycan epitope constructs (e.g., incorporating ganglioside NeuGcGM3) have also been designed with promising overall survival data in studies with incorporation into maintenance-phase regimens (95, 96). Short peptide vaccines, which mimic a key portion of the targeted antigen structure, have been shown to be moderately effective in early studies; and longer peptide motifs of TAAs and immunostimulatory ganglioside targets (e.g., GM3-ganglioside modified peptides) have been used simultaneously to augment CD8+ TIL and CD4+ helper cell responses (97). Employing TAA peptides such as Mucin 1 (MUC1) sequences has been one approach, with a current phase II study for patients with early- and late-stage NSCLC. This vaccine trial (NCT01720836) is paired with a PolyICLC adjuvant, which primes immunity with a TLR-3 ligand to activate DCs (94, 98). Historically, while such advances have also led to vaccines synthesized using long peptides for the treatment of melanoma, as ICI was introduced for advanced disease, experiments have begun to combine PD-1 checkpoint blockade with peptide vaccine platforms. To date, several ongoing trials have paired PD-1/PD-L1 axis blockade simultaneously to empower antigen-sensitized CD8+ T cells in parallel to the vaccine’s antigen delivery platform (87, 93, 94). These combinations have also demonstrated immunologic responses by both CD4+ and CD8+ T cells (99). Intriguingly, a phase III international study now enrolling >300 patients is examining a 9-epitope peptide vaccine (OSE210; which includes a variety of TAAs including MAGE, HER-2, among others in emulsified adjuvant): Treatment with vaccine versus docetaxel is randomized in HLA-A2 positive patients with metastatic NSCLC that have secondary resistance to ICI therapy (NCT06472245) (94). Several peptide vaccines have also been paired with radiotherapy as a novel platform modification that can release tumor antigens from cell injury to drive immunity against distant tumor sites (abscopal effect) (94, 100).

#### 5.1.1 Unique viral vector and mRNA platforms

Vaccine platforms involving viral vectors, including oncolytic viruses as well as nucleic acid (mRNA in particular) delivery methods allow for especially high and long-lasting immunogenicity. While TSA or TAA targeting approaches can be employed in the latter strategy, there is also the theoretical advantage also of avoiding genomic integration risk in mRNA vaccines (87). On the other hand, viral vaccines, including oncolytic virus platforms have a unique advantage of combining innate and adaptive immune responses to viruses while tumor-cell lysis releases cancer-specific antigens (including neo-antigens) to promote long-lasting immunity (101). With greater innovation on this front, the increasing “personalized” approach to vaccine therapeutic development in lung cancer facilitates CD8+ T cell augmented Signal-1 (antigen-specific) responses against a variety of both TSAs and/or TAAs: The combined specificity in the system brings an advance over potent but less specific (e.g., ICI) approaches, while ongoing studies are looking to adopt integration of both modalities (99, 102). In a viral vector phase I/II trial (NCT02879760), MAGE-A3 antigen, expressed in ~ 35-50% of NSCLC tumors, is targeted with an oncolytic viral vaccine that includes Ad/MAGE-A3 and MG1- (Maraba virus) MAGE-A3, designed to only replicate in cancer cells (103). Enrollment includes patients undergoing PD1/PDL1 blockade, and includes phase 2 patients who have progressed after ICI treatment. In the area of mRNA vaccines, two mRNA vaccine trials that have achieved phase II testing (NCT05557591 and NCT00923312) include platforms that test either a panel of TAAs (e.g., MAGE melanoma antigens complexed with either Claudin 6 or Survivin with 5T4 oncofetal antigen, among others (87, 94);). While various phase I trials (reviewed in (87, 94, 103)) continue to enter candidate targets into the pipeline under these unique platforms, specificity and clonal breadth across heterogeneous tumor regions (along with ECM, stromal, tumor-metabolic, and other barriers) will remain challenges that drive the insights ripe for parallel discovery highlighted in relevant sections within this review.

### 5.2 Advancing cell therapies: engineered CD8+ T cells in the dynamic lung cancer antigen landscape

The implementation of Adoptive Cell Therapy (ACT) as an immunotherapy to treat hematologic malignancies or melanoma has brought success, but has not been widely adopted among multiple solid tumor types. ACT immunotherapy broadly encompasses any therapy involving expansion, activation, and sometimes genetic alteration of T cells purified from the host with re-infusion after such *in vitro* modifications (88). In some cases, TIL cells are expanded as the mixed CD8+ and CD4+ T cell population derived directly from the primary tumor. Cells are then IL-2 expanded with or without additional treatments (88, 104). Implementation of ACT has mostly been focused on melanoma, with challenges in applications to other tumors, including lung cancer (105). Nevertheless, recent melanoma studies demonstrate that novel approaches may identify distinct tumor-specific T cell clonotypes associated with clinical responses to TIL-ACT. Identification of these unique T cell clones may identify key approaches for reducing exhaustion of expanded TILs known as “functional reinvigoration” (106, 107). Beyond ex-vivo engineered T cells, methods to boost endogenous dendritic cell functions to keep pace with the variable antigenic landscape of lung carcinoma cells are on the horizon, and may also show promise in the development of novel therapeutic platforms (108). Nevertheless, while this is outside the scope of an engineered/adoptive cell therapy, methodology to boost DC functions such as the quality and magnitude of tumor antigen presentation in parallel would complement ACT approaches outlined herein.

#### 5.2.1 Informing the design of novel T cell receptors in cell therapy: TCR-T engineering

An additional approach to augmenting the function of ex-vivo T cells is to genetically introduce T cell receptors (TCR) that recognize specific lung cancer antigens, or even a family of TCRs detecting TSAs and/or TAAs to T cells collected from any given patient via leukapheresis. For example, such TCR-T therapies often introduce the novel TCR construct(s) via retroviral transduction, while re-infusion of the engineered TCR-T cells is carried out with a non-myeloablative lymphodepleting regimen. Along with treating such cells with recombinant IL-2, this combination creates a favorable state that includes the removal of suppressive cells in the host for the new TCR-T expansion and efficacy. Examples of such trials achieving phase II in lung cancer at this time include constructs targeting the cancer-testis antigen NY-ESO-1 (e.g., NCT01967823) expressed in a subset of NSCLCs as well as melanoma, among other carcinomas. In an alternative strategy, data from key neo-antigen TILs harvested from a patient’s tumor is used to express TCRs from the TIL clones in autologous peripheral blood lymphocytes (PBLs), and re-infuse them into the patient. In one such phase II trial underway (NCT03412877) additional epitopes in the vaccine may also include oncoviral antigens (i.e., HPV if relevant) or mutated oncogene products (e.g., KRAS) while therapy is paired with ICI (e.g., pembrolizumab) (88). Other TCR-T approaches coming online as phase I studies include cancer-testis antigens as targets, and augmented specificity utilizing KRAS mutation-specific TCRs (88, 109).

### 5.3 CAR-T engineering and unique challenges in lung cancer

While ACT approaches also now include acceleration in TCR therapy along with novel NK cell therapies (88), rapid developments in Chimeric Antigen Receptor T (CAR-T) cell therapies have been promising, although met with some major challenges in lung cancer. In particular, the lack of high-level uniformly (clonally) expressed neo-antigens in carcinomas poses one of the greatest challenges in developing a stable and highly expressed target for such engineered T cells (6, 8, 88). In current (mostly phase I) trials in lung cancer, the CAR-T cell’s extracellular single-chain variable fragment (scFv) has been engineered to bind to TAA ligand targets such as CEA, MUC1, or EGFR in NSCLC trials, and DLL3 in SCLC, using mostly autologous cell preparations (88). The intracellular domain of current higher generation CAR-T cells in lung cancer typically includes a key activation domain with one or two co-stimulatory components (e.g., CD28 and OX-40 or 4-1BB) that promote expansion upon target binding. On-target/off-tumor binding to host antigens along with cytokine release syndrome remains a concern and major challenge. Recent constructs include targeting multiple epitopes and higher generation engineering with greater co-stimulatory capacity (107, 110–112). Advances in engineering have introduced hybrid-recognizing CAR-T cells which are currently being evaluated pre-clinically and may enhance therapeutic efficacy through engineering of cell-intrinsic specific cytokines (“armored CAR-T”), or through the inclusion of interleukin expression constructs (e.g., IL-12) integrated into the novel CAR-T products (113, 114).

### 5.4 Boosting dendritic cell responses to empower the CD8+ T cell in lung cancer

Dendritic cells (DCs) are “professional” antigen presenting cells that play important roles in presenting foreign antigens to T cells as part of the adaptive arm of immunity. In carcinomas, tumor cell neo-antigens presented by cross-presenting classical DCs (cDC1 subset) on MHC-I to the cognate TCR of CD8+ T cells elicit among the strongest anti-tumor adaptive immune responses (115). This Signal 1 event on the DC surface is flanked by a robust set of co-stimulatory (along with co-inhibitory) molecules at the immune synapse with the T cell. In lung cancer, these powerful T-effector “educating” cells are capable of migrating to tumor-draining lymph nodes via intrapulmonary collecting lymphatic conduits, where T-effector cells may expand in response to presented and free/apoptotic tumor antigens via afferent lymphatics vessels to secondary (lymph node) and even tertiary lymphoid organs (TLOs) (116). Moreover, resident follicular DCs at these sites may also take up tumor antigens for presentation to resident naïve CD8+ T cells to facilitate parallel anti-tumor immunity.

#### 5.4.1 DC-based immunotherapies and lung cancer

Historically, unique robust host-cell TAAs overexpressed by prostate cancer cells had been an initial area of focus in developing DC based immunotherapy approaches by pulsing ex-vivo DCs with antigen, and inducing DC maturation prior to returning cells to the host (117, 118). The prostate TME can impair T-effector cell and DC functions, allowing tumors to evade immune detection by undermining antigen presentation capabilities of tumor-associated DCs (117, 119). Prostate-specific TAAs such as Prostate-Specific Antigen (PSA) and Prostatic Acid Phosphatase (PAP) may be loaded onto DCs encoded in mRNA to boost anti-tumor cell immune recognition (118). Additionally, DCs engineered to secrete IL-12 or express CD40L may strengthen T-cell activation and improve CD8+ T cell mediated anti-tumor responses known in lung cancer to occur more robustly in tumors with higher neo-antigen burden (8). Several ongoing trials in lung cancer in phase I/II testing are now directly priming DCs ex-vivo with synthetic peptides encoding TAAs or even neo-antigen pulsing of TSAs as DC vaccines with subcutaneous or intradermal delivery (87, 120, 121). Further human studies have also harnessed intra-tumoral injection of DCs engineered to release the chemokine CCL21 that in turn attracts T cells to the TME (19, 122). Delivery in parallel to ICI (i.e., anti-PD-1 antibody) in this setting is being employed to further inhibit tumor apoptotic immunosuppressive forces. Future DC engineering strategies might include novel methods to enhance endogenous antigen presentation functions and thus more efficiently priming endogenous CD8+ T-cells to target tumor cell TSAs while reversing local immunosuppression within the tumor microenvironment (123).

### 5.5 Facilitating cytotoxic T cell engagement with the tumor plasma membrane: a perilous chasm

Homing and presence of anti-tumor sensitized CD8+ T cells to the tumor should be considered a major achievement, and represents the goal of many lung cancer immunotherapy development efforts. However, achieving a last 50–200 nm depth across the tumor cell glycocalyx (67, 68) and docking the T-cell TCR onto a tumor membrane TSA or TAA target (whether presented in the context of MHC-I or not) is yet another feat that is not easily accomplished in the lung carcinoma TME. A variety of novel strategies are underway through ongoing studies (highlighted in (89, 124) to harness antibody technology to engage CD8+ T cells with antigen targets on the lung cancer cell-surface. Such Bispecific T cell engager (BiTE) antibodies are composed of one arm that binds to a major T cell surface component and the other that spans to bind to a major TAA or TSA (125). For example, Tarlatamab is an FDA-approved BiTE that binds to the major TCR co-receptor CD3 while spanning to bind to the DLL3 antigen on small cell lung cancer cells (89, 124). Several ongoing T-cell engager studies in lung cancer include a variety of tumor-cell targets (e.g., EpCAM, B7-H6, Glypican-3, EGFR, among others), often with the T-cell engagement mechanism involving CD3 binding on the T-cell surface (89).

#### 5.5.1 Overcoming tumor-surface glycocalyx repression

A variety of immunosuppressive mechanisms drive NK as well as CD8+ T cell inhibitory and apoptotic signaling upon contacting the glycan-rich layer (glycocalyx) that coats several malignant tumors (57, 60, 61, 68). Heavy sialic acid modification on the termini of tumor-surface glycoproteins and glycolipids that project into the glycocalyx “canopy” (approximately 100–200 nm above the tumor-cell plasma membrane) represses cellular immunity via inhibitory Siglec 7 and 9 receptors on the CD8+ T cell (and NK cell) surface. Expression of sialic acid on gangliosides and as polysialic acid polymers is particularly heavy in SCLC (74), where such expression may also shield ligands for the CD8+ T cell and NK cell activating receptor NKG2D (58, 71). Current strategies to inhibit this lung cancer cell-surface glycocalyx shield and form of CD8+ T cell repression include conjugating sialidase enzyme in a bispecific agent to a tumor-surface target (e.g., PD-L1 or B7-H3). Pre-clinical work is early in development in this novel arena of immunotherapy and glycan immune-checkpoint blockade (61, 70, 72).

## 6 Conclusion

Adaptive immunity and the development of anti-tumor CD8+ cytotoxic T cell responses in carcinoma is critical for long-term control and achieving cure. Achieving this robustly and broadly across the TME is challenging due to a variety of physical barriers, suppressive immunologic responses, and the heterogeneous and dynamic antigen landscape of carcinomas. There are particularly unique limitations in lung cancer, where beyond suppressive forces due to pathologic tumor vasculature and metabolic as well as immunologic mediators of CD8+ T cell inhibition, distinct challenges limit viable immune-cell engagement with the tumor cell membrane. At the same time, lung pathophysiology along with complicating factors in current lung tumor treatment strategies further limit cellular immunity. Avenues to boost and engineer augmented CD8+ T cell responses in lung cancer are evolving quickly, and include novel adoptive cell strategies, advances in peptide, mRNA, viral, and cell-based vaccine approaches; and novel ways to engage critical tumor-surface contacts by activated cytolytic T cells. In pre-clinical research and design, it is instructive to propose novel avenues by which CTLs in lung cancer may overcome stromal and ECM barriers, facilitate or drive gradients by T cell responsive chemokines in the TME, and augment presentation of tumor antigens by empowered antigen presenting cells in the primary tumor and draining lymph nodes (1, 17, 50, 64, 103, 126, 127). **Table 2** highlights such considerations and novel opportunities in light of current limitations and barriers. New insights are advancing our understanding of key homeostatic mechanisms, tumor tolerance, and immune escape mechanisms in lung cancer, fueling many new opportunities for augmenting effector host responses.

**Table 2:** Opportunities for augmenting T-Cell efficacy in carcinoma treatment:.

Major functional or anatomic limitation	Specific hurdle(s) impacting CD8+ T-cell immunity	Potential and emerging avenues for intervention
The tumor vasculature and T cell trafficking	- Imparied flow of a substrate of naïve or prior-sensitized (memory) CD8+ T cells into the tumor parenchyma - Pathologic tumor vasculature and T-cell trafficking limitations across endothelial barriers - Lymphatic remodeling and altered lymphoid organ T-cell trafficking	- Tumor angiogenic vessel pericyte reconstitution (55) - Reducing tumor vascular flow inefficiency (i.e.,”normalization” by VEGF blockade) (64) - Drive spatial expression of effector T-cell “long-distance” chemokine gradients: e.g., DC CCL21 driving CTLs (20, 122) - Restoring sphingosine-1 phosphate (S1P) function for augmented lymph node T-cell traffic kinetics (128)
Extracellular matrix as a lung cancer TME barrier	- Matrix collagen and its proliferation surrounding tumor nests - Matrix hyaluronan structure - Balance of chemokines that promotes tumor-centric drive - Bridging tumor-cell attachment and nano-spatial gaps in tumor nests	- Digesting physical matrix barriers: Enzyme therapeutic considerations (e.g., collagenase) (1, 22) - Alter hyaluronan architecture and fragmentation (21) - Local chemokine modulation: T cell – tumor-cell centric drive (CCL5, CCL21, CXCL9, CXCL10) (20) - Blocking T-cell repression by the tumor glycocalyx (60, 61)
Tumor antigen presentation and recognition: Boosting Signal 1 recognition and immune synapse events	- Limited quantity of neo-epitopes (weak Signal-1 substrate) - Carcinoma tumor heterogeneity and dynamic mutational landscape (antigen poly-clonality and instability) - Antigen presenting cell (APC) paucity and tolerogenic bias - Sensitized CD8+ T cell shielding/suppression by tumor glycocalyx - Immune synapse suppression: A plethora of T-cell “brakes” and paucity of T-cell “accelerators”	- Engineering of bi-functional sialidase and ICI engagement molecules (helping ICI cross the glycocalyx barrier) (61) - ACT: TCR-T & TIL engineering & integration strategies (88, 109) - Optimizing CAR-T constructs: Personalizing target antigens (6, 110–112) - Bispecific engagers to augment TCR - Antigen proximity (89) - Vaccines, including pairing with ICI (87, 103) - Addressing ICI resistance & additional exhaustion targets (LAG3, TIM-3, TIGIT) (50) - Empower immune checkpoint stimulators: e.g., CD40, OX-40, 4-1BB (50, 129)
TME cytokines and favorably tilting the effector – tolerance balance	- Suppression of CD8+ T cells (activation and proliferation) by distinct TME cytokines (stromal, myeloid, and tumor sources) - Inducers of tolerogenic behavior by Dendritic Cells or other APCs (e.g., macrophages) by relevant cytokines - MDSCs and Tregs are drivers of suppressive cytokines in the lung cancer TME	- Local IL-2, IL-12, GM-CSF stimulation: Integration into Vaccines (130) - Limit/inhibit TGF-β, IL-10; M2 macrophage products (131) - Altering suppressor macrophage presence; boosting the M1 subset (131) - Inhibition or depletion of MDSC source-cells: e.g., anti-Gr1, anti-Ly6G; Arg1/ROS inhibition, others (possible anti-PDL1 ICI pairing) (132)
Stromal cell barriers beyond the vascular and immunologic infrastructure	- Cancer-associated Fibroblasts (CAFs) drive T-effector cell inhibitory forces: Stiffening of ECM, secretion of inhibitory cytokines (TGF-β). - Suppressive cancer-associated macrophages (e.g., M2 type) and their products/ICI ligands also block CD8+ T-effector activity.	- Therapeutic inhibition of Fibroblast Activation Protein (FAP); antagonize IL-6/TGF-β drivers of CAF growth with antibody approaches (3, 22) - FAP-directed CAR-T approaches (133) - Inhibiting receptor tyrosine kinases for key growth factors such as FGF-2, PDGF, VEGF, which drive lung tumor fibrotic responses (3) - M2 to M1 re-programming (TLR agonists), M2 depletion using siRNA or miRNA loaded nanoparticles (43, 134)
